# Advances in diffuse glial tumors diagnosis

**DOI:** 10.1055/s-0043-1777729

**Published:** 2023-12-29

**Authors:** Luis Filipe de Souza Godoy, Vitor Ribeiro Paes, Aline Sgnolf Ayres, Gabriela Alencar Bandeira, Raquel Andrade Moreno, Fabiana de Campos Cordeiro Hirata, Frederico Adolfo Benevides Silva, Felipe Nascimento, Guilherme de Carvalho Campos Neto, Andre Felix Gentil, Leandro Tavares Lucato, Edson Amaro Junior, Robert J. Young, Suzana Maria Fleury Malheiros

**Affiliations:** 1 Hospital Israelita Albert Einstein, Departamento de Radiologia, Seção de Neuroradiologia, São Paulo SP, Brazil.; 2 Universidade de São Paulo, Faculdade de Medicina, Hospital das Clínicas, Seção de Neuroradiologia, São Paulo SP, Brazil.; 3 Hospital Israelita Albert Einstein, Laboratório de Patologia Cirúrgica, São Paulo SP, Brazil.; 4 Universidade de São Paulo, Faculdade de Medicina, Departamento de Patologia, São Paulo SP, Brazil.; 5 Instituto do Câncer do Estado de São Paulo, Departamento de Radiologia, Seção de Neuroradiologia, São Paulo SP, Brazil.; 6 Rede D'Or São Luiz, Departamento de Radiologia, Seção de Neuroradiologia, São Paulo SP, Brazil.; 7 Hospital Israelita Albert Einstein, Departamento de Radiologia, Seção de Medicina Nuclear, São Paulo SP, Brazil.; 8 Hospital Israelita Albert Einstein, Departamento de Neurocirurgia, São Paulo SP, Brazil.; 9 Grupo Fleury, São Paulo SP, Brazil.; 10 Memorial Sloan-Kettering Cancer Center, Neuroradiology Service, New York, New York, United States.; 11 Hospital Israelita Albert Einstein, Departamento de Oncologia, Seção de Neuro-Oncologia, São Paulo SP, Brazil.

**Keywords:** Glioma, Neuroimaging, Biomarkers, Genetic Profile, Pathology, Precision Medicine, Machine Learning, Glioma, Neuroimagem, Biomarcadores, Perfil Genético, Patologia, Medicina de Precisão, Aprendizado de Máquina

## Abstract

In recent decades, there have been significant advances in the diagnosis of diffuse gliomas, driven by the integration of novel technologies. These advancements have deepened our understanding of tumor oncogenesis, enabling a more refined stratification of the biological behavior of these neoplasms. This progress culminated in the fifth edition of the WHO classification of central nervous system (CNS) tumors in 2021. This comprehensive review article aims to elucidate these advances within a multidisciplinary framework, contextualized within the backdrop of the new classification. This article will explore morphologic pathology and molecular/genetics techniques (immunohistochemistry, genetic sequencing, and methylation profiling), which are pivotal in diagnosis, besides the correlation of structural neuroimaging radiophenotypes to pathology and genetics. It briefly reviews the usefulness of tractography and functional neuroimaging in surgical planning. Additionally, the article addresses the value of other functional imaging techniques such as perfusion MRI, spectroscopy, and nuclear medicine in distinguishing tumor progression from treatment-related changes. Furthermore, it discusses the advantages of evolving diagnostic techniques in classifying these tumors, as well as their limitations in terms of availability and utilization. Moreover, the expanding domains of data processing, artificial intelligence, radiomics, and radiogenomics hold great promise and may soon exert a substantial influence on glioma diagnosis. These innovative technologies have the potential to revolutionize our approach to these tumors. Ultimately, this review underscores the fundamental importance of multidisciplinary collaboration in employing recent diagnostic advancements, thereby hoping to translate them into improved quality of life and extended survival for glioma patients.

## Resumo

Nas últimas décadas, houve avanços significativos no diagnóstico de gliomas difusos, impulsionados pela integração de novas tecnologias. Esses avanços aprofundaram nossa compreensão da oncogênese tumoral, permitindo uma estratificação mais refinada do comportamento biológico dessas neoplasias. Esse progresso culminou na quinta edição da classificação da OMS de tumores do sistema nervoso central (SNC) em 2021. Esta revisão abrangente tem como objetivo elucidar esses avanços de forma multidisciplinar, no contexto da nova classificação. Este artigo irá explorar a patologia morfológica e as técnicas moleculares/genéticas (imuno-histoquímica, sequenciamento genético e perfil de metilação), que são fundamentais no diagnóstico, além da correlação dos radiofenótipos da neuroimagem estrutural com a patologia e a genética. Aborda sucintamente a utilidade da tractografia e da neuroimagem funcional no planejamento cirúrgico. Destacaremos o valor de outras técnicas de imagem funcional, como ressonância magnética de perfusão, espectroscopia e medicina nuclear, na distinção entre a progressão do tumor e as alterações relacionadas ao tratamento. Discutiremos as vantagens das diferentes técnicas de diagnóstico na classificação desses tumores, bem como suas limitações em termos de disponibilidade e utilização. Além disso, os crescentes avanços no processamento de dados, inteligência artificial, radiômica e radiogenômica têm grande potencial e podem em breve exercer uma influência substancial no diagnóstico de gliomas. Essas tecnologias inovadoras têm o potencial de revolucionar nossa abordagem a esses tumores. Em última análise, esta revisão destaca a importância fundamental da colaboração multidisciplinar na utilização dos recentes avanços diagnósticos, com a esperança de traduzi-los em uma melhor qualidade de vida e uma maior sobrevida.

## INTRODUCTION

Since Virchow's seminal description of neuroglia in the mid-nineteenth century, the diagnostic approach to central nervous system (CNS) tumors has evolved considerably. Initially predicated solely on pathological anatomy, 1 modern methods now incorporate a multidisciplinary framework, fusing traditional morphological assessments with advances in genetics, epigenetics, and molecular oncogenesis.

This review offers a comprehensive overview of the diagnostic landscape for CNS diffuse glial tumors in light of the fifth edition of the WHO Classification of Tumors of the Central Nervous System (WHO CNS5). In particular, we emphasize the critical importance of a multidisciplinary strategy for precise diagnosis, prognosis determination, and therapeutic decision-making. The integration of molecular profiling and cutting-edge technologies has ushered in a new era in neuro-oncology, enabling patient-specific precision medicine and the identification of novel therapeutic targets.

## MAJOR CHANGES IN 2021 WHO CLASSIFICATION, 5 ^TH^ EDITION ( TABLE 1 )

**Table 1:** WHO 2021 classification of diffuse gliomas 2

**Types**	**Adult-type** **Diffuse glioma**	**Pediatric-type** **Diffuse low-grade glioma**	**Pediatric-type** **Diffuse high-grade glioma**
	Astrocytoma, IDH-mutant	Diffuse astrocytoma, MYB- or MYBL1-altered	Diffuse midline glioma, H3 K27-altered
	Oligodendroglioma, IDH-mutant and 1p/19q-co-deleted	Angiocentric glioma	Diffuse hemispheric glioma, H3 G34-mutant
	Glioblastoma, IDH-wildtype	Polymorphous low-grade neuroepithelial tumor of the young	Diffuse pediatric-type high-grade glioma, H3-wildtype and IDH-wildtype
		Diffuse low-grade glioma, MAPK pathway-altered	Infant-type hemispheric glioma

Abbreviations: IDH, isocitrate dehydrogenase; MAPK, mitogen-activated protein kinase; MYB, myeloblastosis gene; MYBL1, myeloblastosis proto-oncogene like 1.

Both clinical and molecular characteristics are instrumental in differentiating adult-type and pediatric-type diffuse glial tumors. Generally, adult-type tumors manifest after age 18, whereas pediatric-type tumors present before this age. However, it is worth noting that adult-type tumors can occasionally occur in children, and conversely, pediatric-type tumors may be observed in adults. 2

Recent advancements in our understanding of tumor molecular biology, particularly concerning isocitrate dehydrogenase (IDH) status, have facilitated the classification of adult-type gliomas into three distinct types: astrocytoma, IDH-mutant; oligodendroglioma, IDH-mutant and 1p/19 qco-deleted; and glioblastoma (GBM), IDH-wildtype. 3 4 The term 'glioblastoma'- now applied only for IDH and H3-wildtype tumors in adult patients- has been expanded to include not only tumors exhibiting classical histological hallmarks, such as necrosis and microvascular proliferation, but also those characterized by telomerase reverse transcriptase (TERT) promoter mutations, epidermal growth factor receptor (EGFR) amplifications, or chromosomal aberrations including gain of chromosome 7 and loss of chromosome 10 (molecularly defined glioblastomas). 5 6

IDH-mutant astrocytomas are now graded from 2 to 4 and separated from IDH-wildtype glioblastomas, thus obsoleting the term “glioblastoma, IDH-mutant.” The grading of astrocytoma may incorporate molecular markers alongside traditional pathological morphology; for example, the presence of cyclin-dependent kinase inhibitor 2A/B (CDKN2A/B) deletion indicates a poorer prognosis and categorizes these tumors as grade 4, even in the absence of microvascular proliferation or necrosis. 7

Oligodendrogliomas, invariably characterized by IDH mutations and co-deletion of chromosomes 1p and 19q, are classified into either grade 2 or grade 3.

Furthermore, the methylation status of O6-methylguanine-DNA methyltransferase (MGMT) has significant therapeutic implications, although it is not included in the classification scheme. Methylation of the MGMT gene promoter, an important DNA repair enzyme, is associated with more potent cell killing and better response to alkylating agents.

Pediatric diffuse gliomas are currently subdivided into eight distinct types: four are low-grade and include diffuse astrocytoma, myeloblastosis gene (MYB) or MYB proto-oncogene like 1 (MYBL1)-altered; diffuse low-grade glioma, mitogen-activated protein kinase (MAPK) pathway-altered; angiocentric glioma; and polymorphous low-grade neuroepithelial tumor of the young (PLNTY). The remaining four types are high-grade and consist of diffuse midline glioma, H3 K27-altered (DMG); diffuse hemispheric glioma, H3 G34-mutant (DHG); diffuse pediatric-type high-grade glioma, H3-wildtype and IDH-wildtype; and infant-type hemispheric glioma. 5

Regarding pediatric-type diffuse glioma, low-grade tumors are classified as grade 1 and high-grade tumors as grade 4. Exceptions include diffuse low-grade glioma, MAPK pathway-altered, and infant-type hemispheric glioma, for which grading has not yet been established. Both diffuse astrocytoma, MYB- or MYBL1-altered, and diffuse low-grade glioma, MAPK pathway-altered, display nonspecific histological features characteristic of low-grade glial tumors and necessitate molecular characterization. These tumors are consistently IDH and H3 wild-type. 8 PLNTY is marked by genetic alterations in the MAPK pathway and features a V-Raf murine sarcoma viral oncogene homolog B1(BRAF) V600E gene mutation in ∼48% of cases; fibroblast growth factor receptors (FGFR) gene fusions may also be present. 9 Angiocentric glioma invariably displays MYB alterations. 2

High-grade DMGs are typified by their midline location and H3-K27 alterations, whereas DHGs are infiltrative gliomas involving the cerebral hemispheres, marked by H3-G34 mutations. Infant-type hemispheric glioma is a high-grade cellular astrocytoma that manifests predominantly in early childhood (<1 year) and is frequently associated with receptor tyrosine kinase (RTK) fusions. 10

Emerging molecular profiles have been integrated into treatment planning for pediatric-type gliomas. For instance, specific BRAF alterations (mutations and fusions) in pediatric-type gliomas and neurotrophic receptor tyrosine kinase (NTRK) family alterations in infant-type hemispheric gliomas can be targeted with molecularly tailored therapies.

## CLINICAL PATHOLOGY AND MOLECULAR-GENETIC TESTS ( TABLE 2 )

**Table 2:** Tumor types, molecular profile, and imaging findings

Tumor type	Molecular/genetic	Imaging
Astrocytoma, IDH-mutant	IDH1, IDH2 mutation, ATRX alteration, p53 mutation, CDKN2A/B codeletion	Relatively homogenous high T2, circumscribed margins, located in the frontal or temporal lobes, T2-FLAIR mismatch.
Oligodendroglioma, IDH-mutant and 1p/19q-co-deleted	IDH1, IDH2 mutation, 1p/19q co-deletion	Supratentorial lesions, frontal lobe, ill-defined margins, and heterogeneous signal on T2, calcification. Enhancement does not correlate well with tumor grade.
Glioblastoma, IDH-wildtype	IDH-wildtype, TERT promoter mutation, EGFR amplification, chromosomes 7 + (gain)/10-(loss)	Heterogeneous enhancement, necrosis, extensive perilesional edema, hemorrhage. Atypical absent enhancement and necrosis in molecularly defined glioblastoma.
Diffuse astrocytoma, MYB- or MYBL1-altered	MYB, MYBL1 alterations	Non-specific imaging features.
Angiocentric glioma	MYB::QKI gene fusion	Ill-defined margins, high T2 with cyst, high T1 areas.
Polymorphous low-grade neuroepithelial tumor of the young (PLNTY)	BRAF p.V600E mutation, FGFR2 or FGFR3 fusions	Well-defined margins, located in the posterior inferior temporal lobe, extensively calcified.
Diffuse low-grade glioma, MAPK pathway-altered	BRAF p.V600E mutation, FGFR1 alterations	Non-specific imaging features.
Diffuse midline glioma, H3 K27-altered	H3 K27 alteration, EGFR mutation (exon 20), EZHIP, ACVR1, PDGFRA	Brainstem, thalamic (sometimes bi-thalamic), or spinal cord locations. High T2 with variable enhancement.
Diffuse hemispheric glioma, H3 G34-mutant	H3 G34-mutation, TP53 mutation, ATRX alteration	Supratentorial hemispheric, restricted diffusion, may show enhancement and necrosis.
Diffuse pediatric-type high-grade glioma, H3-wildtype and IDH-wildtype	H3-wildtype, IDH-wildtype	Non-specific aggressive imaging features (e.g., necrosis, restricted diffusion).
Infant-type hemispheric glioma	NTRK family, ROS1, ALK, or MET fusions	Non-specific aggressive imaging features (e.g., necrosis, restricted diffusion).

Abbreviations: ACVR1, activin A receptor type I; ALK, anaplastic lymphoma receptor tyrosine kinase; ATRX, α thalassemia/mental retardation syndrome X-linked; BRAF, V-Raf murine sarcoma viral oncogene homolog B1; CDKN2A/B, cyclin-dependent kinase inhibitor 2A/B; EGFR, epidermal growth factor receptor; EZHIP, EZH inhibitory protein; FGFR, fibroblast growth factor receptors; IDH, isocitrate dehydrogenase; MET, MET proto-oncogene receptor tyrosine kinase; MAPK, mitogen-activated protein kinase; MYB, myeloblastosis gene; MYBL1, myeloblastosis proto-oncogene like 1; NTRK, neurotrophic tyrosine receptor kinase; PDGFRA, platelet-derived growth factor receptor α; ROS1, ROS proto-oncogene 1 receptor tyrosine kinase; TERT, telomerase reverse transcriptase.

**Figure 1:**
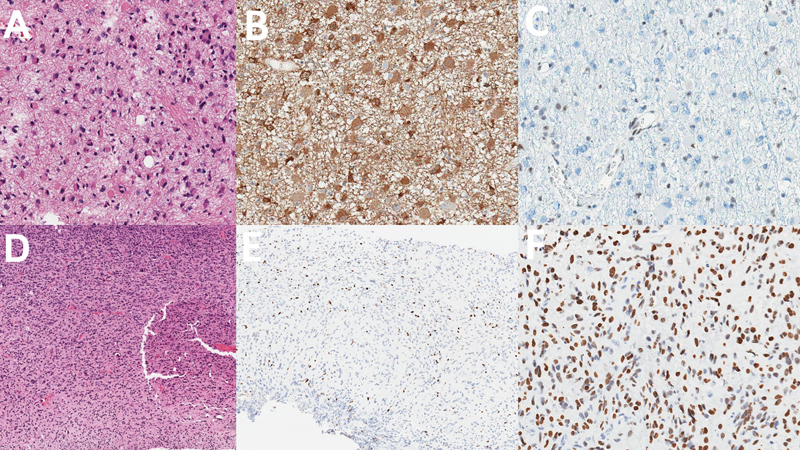
Two examples of different glial neoplasms defined by molecular findings. The first case ( **A-C** ) is a hemispheric tumor from a 43-year-old male patient. The cells have typical astrocytic morphology ( **A** ), with immunohistochemistry positive for IDH1 R132H ( **B** ) and ATRX loss ( **C** ). These findings are consistent with astrocytoma, The second case ( **D-F** ) is a pontine tumor in a 8-year-old patient. Although morphology is also astrocytic ( **D** ), the loss of H3K27me3 expression ( **E** ) and positivity for H3K27M mutation ( **F** ) rendered this patient a “diffuse midline glioma, H3 K27-altered” diagnosis.

Diffuse gliomas are characterized in diagnostic pathology by a growth pattern of individual tumor cells growing through the brain parenchyma, as opposed to the sharp pushing border of circumscribed astrocytic tumors or brain metastasis. 2 The morphological pattern can disclose high-grade features (such as necrosis, microvascular proliferation, and/or mitotic figures) or suggest possible molecular changes (as in adult-type oligodendroglioma, defined by IDH mutations associated with 1p/19q co-deletion) ( Figure 1 ).

Molecular profiling plays a pivotal role in WHO CNS5. Various genetic alterations can be identified using immunohistochemistry (IHC) surrogate assays, such as IDH1 R132H, ATRX, p53, BRAF V600E, H3K27M, H3K27me3 and H3 G34R/V. Others, including CDKN2A/B homozygous deletion, EGFR amplification, and 1p/19q co-deletion, can be detected using fluorescence in situ hybridization (FISH). Assessment of O6-methylguanine-DNA methyltransferase (MGMT) methylation status is feasible through polymerase chain reaction (PCR) and pyrosequencing techniques. 11

In certain instances, immunohistochemistry alone may be insufficient for a comprehensive diagnostic categorization. For example, in patients who test negative for the IDH1 R132H mutation by IHC, additional molecular testing should be performed if a patient is younger than 55 years old; shows ATRX-loss or oligodendroglial morphology. Alternative methodologies, such as next-generation sequencing or methylation profiling, are required for this purpose. In contrast, patients >55 years old who have grade 4 gliomas that test negative for the IDH1 R132H mutation can be assumed also to be wildtype for the less common IDH variants and declared to have IDH-wildtype glioblastoma WHO grade 4.

Next-generation sequencing (NGS) can elucidate a myriad of genetic alterations, including but not limited to non-canonical IDH1 and IDH2 mutations, and NTRK fusions; these mutations have potential targeted therapies. 12 13 Although limited by its higher cost, DNA- and RNA-based NGS can detect multiple genetic alterations in a single test without needing a “multistep” approach that can lead to excessive tissue degradation.

Advancements in DNA methylation profiling have also improved the classification of brain tumors. This technique enables the quantitative assessment of selected methylation sites across the genome, greatly enhancing diagnostic precision. Notably, it has also led to identifying previously unknown tumor subtypes and helping in cases with discordant clinical, morphological, and immunophenotypical patterns. 6 Most tumor types in WHO CNS5 have a distinct methylation signature, and some rare tumor types and subtypes can only be identified by methylation profiling. Despite this, there are still caveats in methodological approaches, threshold used, and the technology is not widely available. 5

## STRUCTURAL IMAGING: MAGNETIC RESONANCE IMAGING (MRI) AND COMPUTED TOMOGRAPHY (CT)

Imaging plays a critical role in the diagnosis and management of tumors, serving as an indispensable tool for pre-operative planning. When performed and interpreted by experienced professionals, imaging can yield a narrowed differential diagnosis contextualized by patient age and symptomatology. Initial decisions on whether to pursue close follow-up, conduct a stereotactic biopsy, or proceed with maximal safe resection are predominantly based on clinical findings and magnetic resonance imaging (MRI).

Recent advancements have enabled the correlation between the genetic/molecular profile and the radiographic phenotype of adult-type diffuse gliomas. For example, IDH-mutant gliomas, encompassing both astrocytoma and oligodendroglioma, predominantly occur in individuals under the age of 55 and are more frequently localized in the frontal lobe and insula. 14 The “T2-FLAIR mismatch” sign stands as the most specific imaging marker for distinguishing IDH-mutant astrocytoma from IDH-mutant, 1p/19q co-deleted oligodendroglioma and IDH-wildtype glioblastomas. This sign has exhibited 100% specificity in some studies and up to 37% sensitivity in diagnosing adult-type diffuse astrocytoma with an IDH mutation. 15 16 17 Although the sign exhibits high specificity, false-positive “T2-FLAIR mismatch” findings have been reported, particularly in pediatric-type and glioneuronal tumors, 18 19 making it an unreliable predictor of IDH status in the pediatric population. Proper observer training is also important to reduce false-positive results. 20 Diffuse astrocytoma WHO grade 2 typically manifests with well-defined borders and relatively uniform high signal intensity on T2 imaging, accompanied by minimal or absent enhancement. 21 Grade 2 adult-type astrocytoma inexorably transforms to grade 3 and 4 over time, developing areas of contrast enhancement and necrosis within the tumor, looking aggressive on imaging. In patients initially presenting with this imaging signs of high aggressiveness (necrosis, hemorrhage, edema), an area of FLAIR signal suppression within the non-enhancing portion of the tumor is a clue to the IDH-mutation status of the lesion. 22

**Figure 2:**
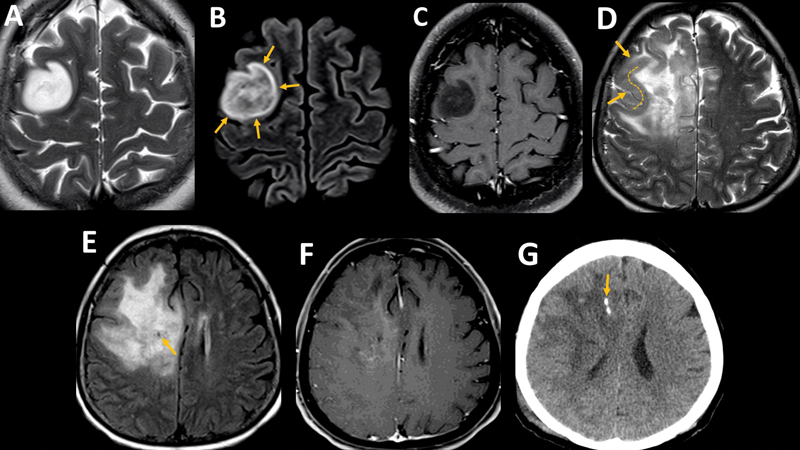
Low-grade adult-type gliomas. Astrocytoma, IDH-mutant in a 29-year-old woman ( **A** , **B** and **C** ). Axial T2( **A** ) shows a relatively homogenous hyperintense lesion on the right middle frontal gyrus. Axial FLAIR ( **B** ) shows a heterogeneous low signal intensity, except for a peripheral rim (arrows) of preserved hyperintensity (T2-FLAIR “mismatch” sign). Axial T1 post-gadolinium ( **C** ) shows no enhancement. Oligodendroglioma, IDH-mutant, 1p/19q co-deleted in a 58-year woman ( **D** , **E** , **F** and **G** ). Axial T2 ( **D** ) demonstrates a heterogeneous hyperintense lesion in the right frontal lobe, with indistinct margins and the sinuous wave-like intratumoral-wall sign, representing cortical thickening (arrows and dashed line) around a T2 heterogeneously hyperintense core. Axial FLAIR ( **E** ) demonstrates no significant signal suppression except for a small cyst (arrow). Axial T1 post-gadolinium ( **F** ) shows no significant lesion enhancement. Axial CT ( **G** ) shows a linear calcification within the lesion (arrow).

In contrast, IDH mutant 1p/19q co-deleted oligodendrogliomas exhibit indistinct margins, signal heterogeneity, significant cortical involvement, the sinuous wave-like intratumoral-wall (SWITW) sign, calcifications, and cysts 21 23 ( Figure 2 ). Calcifications and extensive cortical involvement with a SWITW sign have demonstrated high specificity (ranging from 90% to 97%) for diagnosing adult-type diffuse oligodendrogliomas. 15 21 23

**Figure 3:**
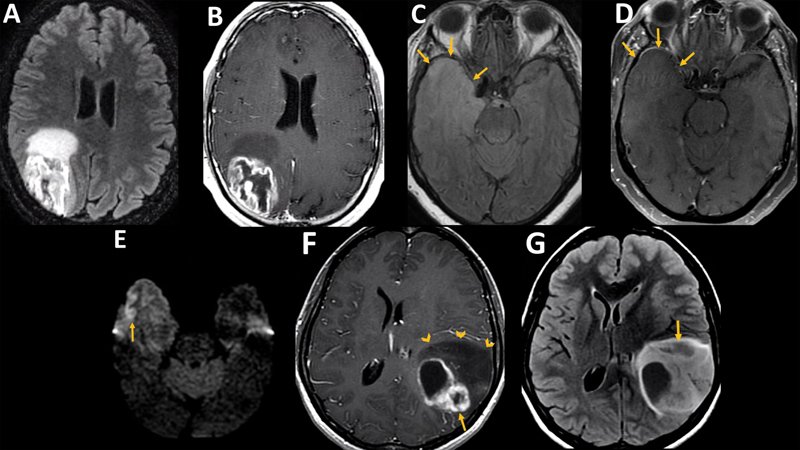
High-grade adult-type glioma. Common GBM presentation in a 52-year-old male ( **A** and **B** ). Axial FLAIR ( **A** ) shows a heterogeneous lesion involving the right parietal and occipital transition, with surrounding edema. Axial T1 post-gadolinium( **B** ) demonstrates thick, irregularly shaped enhancement with central necrosis. Uncommon GBM presentation in a 66-year-old female ( **C** , **D** , and **E** ). Axial FLAIR ( **C** ) shows an infiltrative lesion involving the right temporal lobe (arrows). Axial T1 post-gadolinium ( **D** ) shows no enhancement in the lesion (arrows). Axial DWI ( **E** ) demonstrates an area of restricted diffusion delineating the cortex (arrow). This patient presented with seizures and was initially treated for herpes encephalitis, but subsequent progression and pathology (not shown) confirmed a GBM. Grade 4 astrocytoma, IDH-mutant in a 34-year-old female ( **F** and **G** ). Axial T1 post-gadolinium ( **F** ) shows a left parietal lesion with contrast enhancement and necrosis (arrow), with a significant non-enhancing component (arrowheads). In the nonenhancing part of the lesion, FLAIR signal suppression is consistent with IDH mutation.

IDH-wildtype GBM usually appears in individuals over 55, presenting as an enhancing lesion with central necrosis, hemorrhage, and surrounding peritumoral edema. 24 Less frequently, these tumors may exhibit minimal or absent contrast enhancement, lack central necrosis, and extensive cortical involvement (molecularly defined GBM) 25 ( Figure 3 ). Locations often include deep regions, particularly the subventricular white matter around ventricular horns and atria, although peripheral locations are also possible.

Risk stratification grounded in reproducible features of conventional morphological imaging has shown a fair correlation with the molecular profile in adult-type diffuse gliomas. 26 In scenarios where genetic/molecular testing is either unavailable or inconclusive for a specific molecular categorization, gliomas are designated by their morphological histopathology as “not otherwise specified” (NOS), such as diffuse astrocytoma, NOS. Prognostication based on MRI findings remains feasible and may be helpful even in these NOS-classified patients. 27

**Figure 4:**
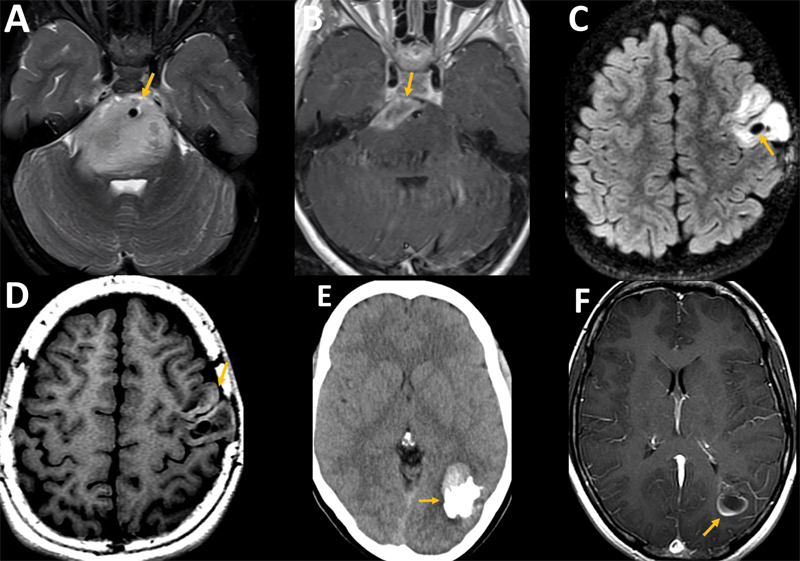
Pediatric type tumors. High grade. DMG in a 3-year-old boy ( **A** and **B** ). Axial T2 ( **A** ) shows an infiltrative lesion centered on ventral pons, extending anteriorly to “embrace” the basilar artery (arrow). Axial T1 post-gadolinium ( **B** ) shows ring enhancement at the right anterior portion of the tumor (arrow). Low Grade. Angiocentric glioma in a 17-year-old boy ( **C** and **D** ). Axial FLAIR ( **C** ) demonstrates a hyperintense lesion with cysts (arrow) on the left middle frontal and pre-central gyrus. Axial T1( **D** ) without gadolinium shows typical linear cortical hyperintensity (arrow). PLNTY in a 18-year-old girl ( **E** and **F** ). Axial CT ( **E** ) demonstrates a massively calcified lesion in the left occipital and temporal lobe transition (arrow). Axial T1 post-gadolinium ( **F** ) shows partial ring-like enhancement (arrow).

Pediatric high-grade diffuse gliomas have some distinct imaging characteristics. Diffuse Midline Glioma, H3-K27 altered (DMG), primarily manifests in the brainstem, thalamus, and spinal cord, often presenting with a rapid onset of symptoms (usually less than three months). 28 The term Diffuse Intrinsic Pontine Glioma (DIPG) describes a specific radiological phenotype—an expansive lesion primarily centered in the ventral pons, occupying more than 50% of the cross-sectional area of the pons in at least one T2 axial image. These lesions may show focal or ring-like enhancement but are usually not entirely enhancing. Notably, the tumor tends to grow ventrally, enveloping the basilar artery. An average of 80% of typical DIPGs in children are molecularly classified as DMG 29 30 ( Figure 4 ). It is worth noting that midline gliomas in adults represent DMG in 15–60% of cases, most frequently located in the thalamus. 31 32 The remaining minority of DIPGs in children without H3-K27 alteration may represent other types of tumors, including low-grade MYB-altered gliomas, high-grade H3-wildtype and IDH-wildtype gliomas, or even adult-type IDH-mutant gliomas. Diffuse Hemispheric Glioma (DHG) and infant-type hemispheric gliomas often exhibit non-specific yet aggressive imaging features, including heterogeneous enhancement with central necrosis, restricted diffusion, hemorrhage, mass effect, and edema.

Diffuse low-grade pediatric gliomas may also have recognizable imaging patterns. Polymorphous Low-grade Neuroepithelial Tumor of the Young (PLNTY) typically presents, in the context of epilepsy, as a well-circumscribed lesions, mainly located in the posterior inferior temporal lobe, and frequently shows dense calcification and cystic components. 9 The prominent calcification is a distinct feature, although one can also account for ganglioglioma in differential diagnosis. Angiocentric gliomas often display ill-defined margins and lack enhancement. A unique feature of these tumors is a cortical or subcortical rim of high signal on T1-weighted imaging. Additionally, a stalk-like extension to the adjacent ventricle on T2 images can be present. Mild atrophy in the surrounding parenchyma can be misinterpreted as sequela ( Figure 4 ).

Diffuse low-grade gliomas that are MAPK pathway-altered, or MYB- or MYBL1-altered, generally exhibit non-specific features characteristic of low-grade tumors. These include non-restricted diffusion, minimal enhancement, and relatively low mass effect relative to the tumor size. Imaging, in this case, has limitations when it comes to distinguishing these specific pediatric lesions from other types, such as circumscribed astrocytic tumors, glioneuronal tumors, or even adult-type tumors. 8

## FUNCTIONAL IMAGING: PERFUSION-WEIGHTED IMAGING (PWI), MRI SPECTROSCOPY (MRS), POSITRON EMISSION TOMOGRAPHY (PET), DIFFUSION TENSION IMAGING (DTI), AND FUNCTIONAL MRI (FMRI)

Perfusion-weighted imaging (PWI) with dynamic susceptibility contrast (DSC) offers a valuable methodology for assessing the aggressiveness of adult-type diffuse gliomas at the time of diagnosis, guiding targeted biopsies, and differentiating between tumor progression and treatment effects. Elevated relative cerebral blood volume (rCBV) is expected in high-grade and IDH-wild type tumors at diagnosis 33 34 35 as well as during post-treatment progression, 36 which is consistent with underlying processes of neoangiogenesis and increased vascularization. However, IDH-mutant 1p/19q co-deleted oligodendrogliomas may show high rCBV without the same negative connotations due to extensive internal vascular networks. 35 37 Dynamic contrast enhancement (DCE) permeability imaging serves as a relevant tool in evaluating post-treatment outcomes versus actual tumor progression. 36 Existing literature exhibits a high degree of accuracy—sensitivity and specificity, reaching 90% and 88% for DSC and 89% and 85% for DCE, respectively. 38 Nevertheless, cross-institutional application of threshold values remains challenging due to significant variability in methodologies, thresholds, and histopathological criteria. Therefore, rigorous standardization and additional scientific validation are imperative prior to widespread clinical adoption of a given threshold value.

Magnetic resonance spectroscopy (MRS) can detect IDH mutations in gliomas with high specificity by identifying the presence of 2-hydroxyglutarate (2HG). 39 40 41 This may be particularly useful in areas with limited access to next-generation testing for full IDH profiling or for tumors in difficult-to-access locations such as the brainstem. Despite its potential, the routine clinical application of 2HG detection via MRS is hindered by a multitude of technical factors. 2HG MRS has low sensitivity for small tumors. 42 It is prone to artifacts, requires local expertise and is technically demanding, hence 2HG MRS is not widely available at large brain tumor centers. Nevertheless, MRS remains useful for distinguishing between low- and high-grade tumors, directing biopsy targets, and differentiating treatment effects from tumor progression. 43

Hybrid positron emission tomography (PET) imaging employs various radiopharmaceuticals to evaluate gliomas, furnishing specific metabolic data that is complemented by either computed tomography (PET/CT) or magnetic resonance imaging (PET/MRI). This multimodal approach is employed for biopsy guidance, delineation of tumor volume, radiotherapy planning, therapeutic response assessment, and discernment between pseudoprogression and relapse. 44 The primary tracers include the glucose analog 18F-fluoro-deoxy-glucose (FDG) and radiolabeled amino acids such as 18F-fluoro-ethyl-L-tyrosine (FET), 11C-methionine (MET), and 6-[18F] Fluoro-L-DOPA (F-DOPA). 44 Although FDG is a cornerstone tracer in oncology, its utility in glioma imaging is constrained by high physiological uptake in normal brain tissue. Conversely, radiolabeled amino acids, which do not exhibit physiological uptake in normal brain tissue, rely on the overexpression of L-type amino acid transporters. A recent meta-analysis contrasted PET imaging and MR perfusion for differentiating treatment-related abnormalities from tumor progression in gliomas, revealing sensitivities of 86% for FDG-PET and 92% for DSC perfusion, with specificities of 85% and 67%, respectively. 45 Despite inherent technological differences, the performance metrics between PET imaging and PWI proved to be relatively congruent, with no statistically significant differences noted across varying PWI techniques and PET tracers.

DTI-based tractography (DTI-tractography) employs diffusion tensor imaging to reconstruct white matter fibers by evaluating tissue diffusivity and directional eigenvectors. Typically, deterministic algorithms are deployed for three-dimensional and multiplanar reconstructions of these fibers. In the surgical setting, DTI-tractography can aid in the selection of surgical corridors and contribute to informed patient counseling by better evaluating the risks associated with tumor resection prior to the procedure and estimating the extent of resection. DTI-tractography can be incorporated with neuronavigation softwares and, when used in conjunction with direct electrical stimulation for subcortical mapping, has been empirically shown to reduce surgical time, minimize the number of stimulations, and lower the risk of intraoperative seizures. 46 47 However, this technique is not without limitations. For example, a single tensor model can resolve only one fiber direction within an imaging voxel, yet white matter voxels frequently comprise multiple fiber orientations. Further, it is constrained by compromised fiber reconstruction in regions of edema or in the presence of susceptibility artifacts, such as blood products or calcifications, particularly near the skull base. Non-tensor tractography methods have been developed to surmount the issue of multidirectional crossing fibers but have yet to gain widespread clinical acceptance. 48

**Figure 5:**
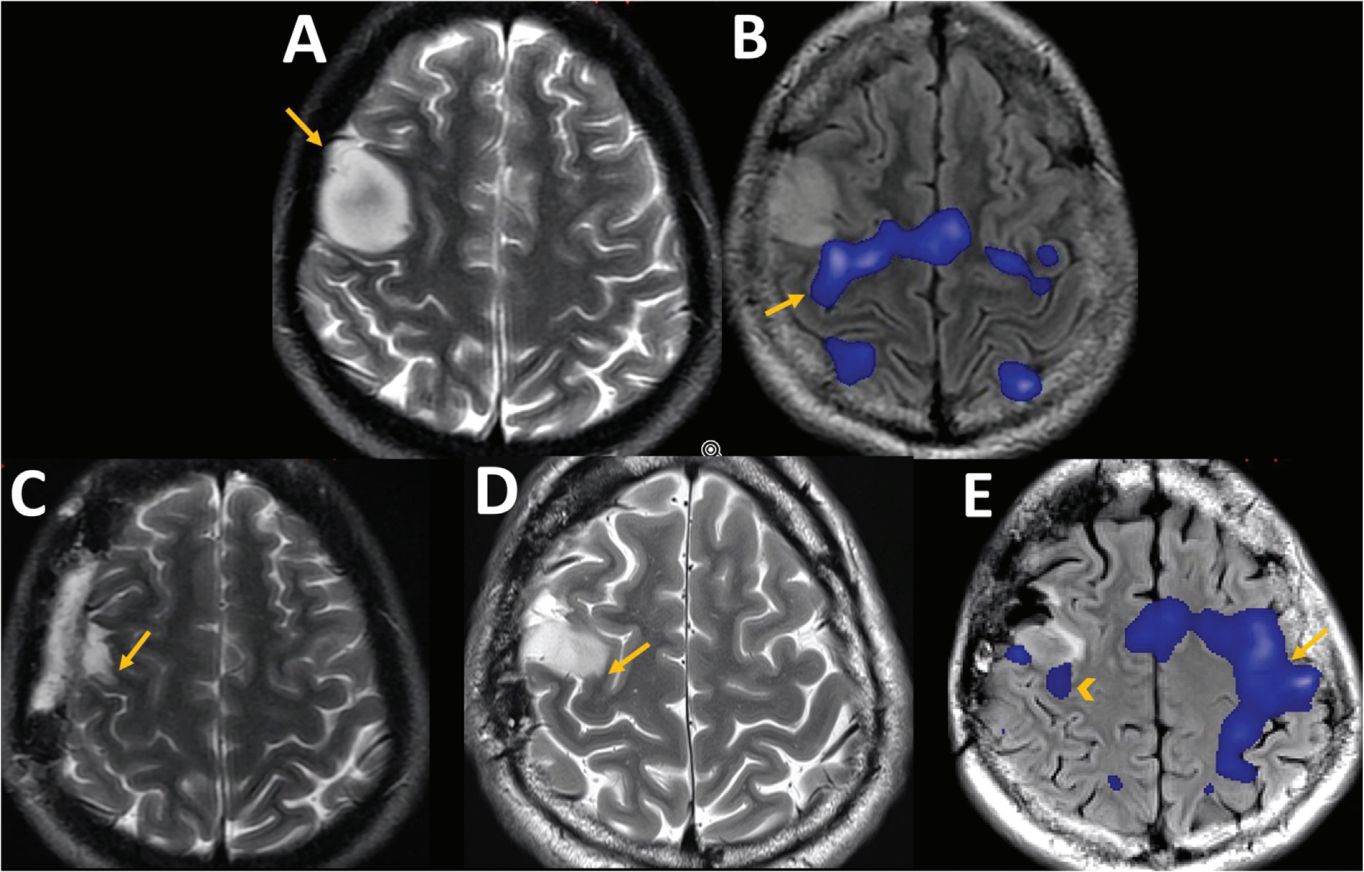
Neuroplasticity over 13 years. Axial T2 ( **A** ) shows an IDH-mutant astrocytoma in the right primary motor cortex (arrow). Axial FLAIR fused with fMRI ( **B** ) with left motor hand task shows BOLD signal in the expected location at the right pre-central gyrus, near the posterior and medial tumoral margin (arrow). Axial T2 ( **C** ) performed five months after near total resection demonstrated only slight hyperintensity at the medial border of the surgical cavity (arrows). Axial T2 ( **D** ), 13 years later, shows a progression of the astrocytoma, now infiltrating medially the pre-central gyrus, toward the hand motor knob area (arrow). At this time, fMRI ( **E** ) with left-hand motor activity shows little BOLD signal in the right pre-central gyrus (arrowhead) but a more extensive BOLD signal in left motor areas (arrow), indicating the recruitment of ipsilateral motor cortex due to neuroplasticity.

fMRI indirectly measures neuronal activity through blood oxygen level-dependent (BOLD) signals based on the principle of neurovascular coupling. This posits that a hemodynamic response is intrinsically linked to neuronal activity. fMRI is versatile and can be employed for a range of cognitive tasks, albeit with varying degrees of success. Sensory-motor and language mapping tasks remain the predominant applications in presurgical planning. 49 Furthermore, fMRI is instrumental in revealing long-term plasticity and the recruitment of alternate brain regions following surgical interventions for tumors located in or near eloquent brain areas 50 ( Figure 5 ). Despite its utility, fMRI is not devoid of limitations. It necessitates a high degree of patient cooperation, can be influenced by altered brain hemodynamics near tumoral tissue, is relatively time-intensive, and requires a multidisciplinary approach coupled with specialized expertise for effective execution. Resting-state fMRI is a newer technique that measures spontaneous fluctuations in BOLD signal in patients while at rest. While promising in patients who are unable to perform task-based fMRI, the methods of analysis to detect intrinsic networks remain heterogeneous and this remains a mostly research tool.

## ARTIFICIAL INTELLIGENCE, MACHINE LEARNING, RADIOMICS, AND RADIOGENOMICS

Artificial Intelligence (AI) broadly encompasses computational tasks that emulate human cognitive functions. Deep learning techniques are already widely used in imaging acquisition to acquire MRI data faster, at higher resolution, and with fewer artifacts. A subset of AI, Machine Learning (ML), employs algorithms that adapt and learn from existing datasets to predict outcomes in new data without explicit programming. The medical domain, particularly diagnostic imaging, has seen accelerated adoption of various ML algorithms, showing substantial promise for advancing precision medicine and therapeutics. 51

Radiomics, an emerging interdisciplinary field that integrates quantitative imaging and ML algorithms, has found extensive applications in cancer imaging. This approach allows for the non-invasive extraction of features from medical images that may be imperceptible to the human eye. Utilizing advanced bioinformatics tools, radiomics quantitatively identifies, extracts, and categorizes a plethora of imaging features. Such extracted data have been instrumental in augmenting diagnostic and prognostic accuracy, identifying genomic alterations in tumor DNA and RNA from CT and MRI images. 52 The confluence of radiographic and genomic data has given rise to a new domain termed 'radiogenomics' 53 . Furthermore, radiomics has the potential to revolutionize oncology by enabling the tailored selection of treatment regimens, as recent research has demonstrated correlations between imaging features and tumor responsiveness to specific therapeutics. 54

Regarding glioma diagnosis, a systematic review and meta-analysis by Van Kempen et al. revealed a broad performance spectrum of ML algorithms in classifying molecular features of gliomas via MRI. 55 Sensitivity and specificity metrics for IDH status varied considerably, with values ranging from 54% to 98% and 67% to 99%, respectively. For the 1p/19q co-deletion characterization, sensitivity and specificity ranged between 68% and 92% and 71% and 85%, respectively.

Despite the burgeoning prospects of AI and ML in medical imaging, several obstacles impede their clinical adoption. One key challenge is the requirement for greater understanding and confidence within the radiology community, where the correct implementation of quantitative imaging is crucial for generating reliable biomarkers. 56 Algorithms require extensive multi-institutional testing and validation to ensure generalizability over heterogeneous imaging data and diverse patient populations Moreover, the seamless integration of these technologies into clinical workflows, coupled with ongoing deliberations surrounding their legal and ethical implications, represents additional hurdles for the responsible deployment of AI technologies in medical practice. 57 58

In conclusion, we are witnessing remarkable advances in glial tumor diagnosis, significantly improving our ability to classify these tumors more accurately. Our diagnostic and prognostic capabilities have grown substantially due to advances in tumor imaging and clinical pathology/genetics. Integrating artificial intelligence and machine learning algorithms has shown promising results in streamlining the interpretation of complex data to provide accurate, non-invasive diagnosis.

These advancements enhance our understanding of the underlying biology and genetics of diffuse glial tumors and pave the way for personalized treatment strategies. Improved diagnostic accuracy enables tailored therapies to the specific molecular profiles of individual tumors, leading to more effective and targeted interventions.

Nevertheless, there are still some disparities in the availability of diagnostic techniques. For example, molecular/genetic tests like immunohistochemistry, PCR, and genetic sequencing are less accessible, especially in the public health systems of low-income countries, while imaging is relatively more accessible. This highlights the importance of developing reliable imaging biomarkers and universally accessible non-invasive approaches.

Interdisciplinary collaboration among neurosurgeons, oncologists, radiologists, nuclear medicine specialists, pathologists, engineers, and data scientists is essential. By working together, we can harness these breakthroughs to provide patients with the best possible care, moving closer to a future where the burden of diffuse glial tumors is minimized, and the prospects for those affected are significantly improved.
